# Altered Interoceptive Perception and the Effects of Interoceptive Analgesia in Musculoskeletal, Primary, and Neuropathic Chronic Pain Conditions

**DOI:** 10.3390/jpm10040201

**Published:** 2020-10-29

**Authors:** Daniele Di Lernia, Marco Lacerenza, Vivien Ainley, Giuseppe Riva

**Affiliations:** 1 Department of Psychology, Università Cattolica del Sacro Cuore, Largo Gemelli, 1, 20100 Milan, Italy; giuseppe.riva@unicatt.it; 2 Humane Technology Lab., Università Cattolica del Sacro Cuore, Largo Gemelli, 1, 20100 Milan, Italy; 3 Neurology and Pain Center, Humanitas San Pio X Clinic, 20159 Milan, Italy; lacerenzam@gmail.com; 4 Lab of Action and Body, Royal Holloway University of London, Egham TW20 0EX, UK; Vivien.Ainley@rhul.ac.uk; 5 Applied Technology for Neuro-Psychology Lab, IRCCS Istituto Auxologico Italiano, Via Magnasco, 2, 20149 Milan, Italy

**Keywords:** chronic pain, interoception, interoceptive treatment, C-Touch, anxiety and depression

## Abstract

Chronic pain (CP) severely disrupts the daily life of millions. Interoception (i.e., sensing the physiological condition of the body) plays a pivotal role in the aetiology and maintenance of CP. As pain is inherently an interoceptive signal, interoceptive frameworks provide important, but underutilized, approaches to this condition. Here we first investigated three facets of interoceptive perception in CP, compared with pain-free controls. We then introduce a novel interoceptive treatment and demonstrate its capacity to reduce pain severity in CP, potentially providing complementary analgesic treatments. Study 1 measured interoceptive accuracy, confidence and sensibility in patients (N = 60) with primary, secondary musculoskeletal, and neuropathic CP. Compared with matched controls, CP participants exhibited significantly lower interoceptive accuracy and interoceptive confidence. Pain severity was predicted positively by interoceptive accuracy, anxiety and depression, and negatively by interoceptive confidence. Study 2 tested a promising new interoceptive treatment for CP, in a single-blind between-subjects design (N = 51) with primary, secondary musculoskeletal, and neuropathic CP patients. The treatment specifically activates the C-Tactile system, by means of controlled stimulation of interoceptive unmyelinated afferents, at 3 cm/s with a force of 2.5 mN. This treatment led to significant pain reduction (mean 23%) in the CP treatment group after only 11 min, while CP controls who received comparable but non-interoceptive stimulation reported no change in pain intensity. These studies highlight the importance of interoceptive approaches to CP and demonstrate the potential of this novel method of C-Tactile stimulation to provide complementary analgesic treatments.

## 1. Introduction

Chronic Pain (CP) is a condition that originates from various pathophysiological mechanisms [1,2] and can be defined as a state persisting for at least three months or beyond the expected time for healing [3]. CP patients exhibit altered processing across various systems including emotion regulation [4,5], cognition [6,7] and memory [8]. Such differences also extend to the processing of interoceptive signals [9].

Pain is inherently interoceptive [10], where interoception is defined as “the process by which the nervous system senses, interprets, and integrates signals originating from within the body, providing a moment-by-moment mapping of the body’s internal landscape across conscious and unconscious levels” [11]. Disrupted interoceptive processing is fundamental to the perception, modulation and chronification of pain [12,13,14], on both cortical [15,16,17] and behavioural levels [9]. In CP research, interoception is generally assessed behaviorally by cardiac ”interoceptive accuracy” [18], i.e., accurate perception of one’s heartbeat [19]. Individuals with complex regional pain syndrome (CRPS) [20], fibromyalgia [21], and multisomatoform CP disorders [22] all exhibit low interoceptive accuracy. However, interoception is not unitary but has several dissociable facets [23], including ”interoceptive confidence” in one’s perceptions (IAconf) and “interoceptive sensibility”, i.e., scores on self-report questionnaires, which are designed to indicate how well people believe that they can feel their interoceptive sensations (IAs) [19], as well as interoceptive accuracy (IAcc) described above. Nevertheless, despite preliminary evidence for low interoceptive accuracy in CP, no study has previously compared measures for several different facets of interoception across different CP conditions.

Importantly, the interoceptive system is also involved in innate analgesic mechanisms, which rely on specific stimulation of the peripheral C-Tactile (CT) afferent system. This system is composed of free tactile arborizations on the superficial layer of non-glabrous skin [24], forming a secondary touch system [25,26,27] that is interoceptive rather than purely somatosensory [28], with implications for affective touch and social bonding [29,30], stress, arousal [31,32] and hormonal modulation [33]. CT receptors respond uniquely to low-force, low-velocity stimuli (2.5 mN, 3 cm/s), being unresponsive to mechanical vibration, high velocities or indentation force [24,29,31]. Crucially, stimulation of the CT system reduces experimentally-induced heat pain in healthy participants [34,35,36,37] and mechanical and chemical pain in animals [38,39]. Mechanisms of CT analgesia are not yet fully understood but potentially relate to: inhibition at the level of the dorsal horn [39]; oxytocin and µ-opioids modulation [33,40]; and autonomic and parasympathetic enhancement [31,41]. CT stimulation thus represents a promising treatment for pain. However, to the best of our knowledge, it has never been tested with CP patients.

In Study 1, we investigated three facets of interoception in CP patients and pain-free controls, hypothesizing that interoceptive measures would be compromised in CP [42] and would predict pain severity. As both CP [4,5] and interoception [43,44] are linked to anxiety and depression, these were also measured.

In Study 2, we tested the effect of interoceptive CT stimulation on CP patients, in a single-blind between-subject design, hypothesizing that interoceptive stimulation would reduce pain severity compared to control stimulation that is non-interoceptive.

## 2. Materials and Methods Study 1

To explore the relationship between CP and facets of interoception, Study 1 compared three measures of interoception (IAcc, IAconf, and IAs) in CP patients versus age- and sex-matched pain-free controls, together with pain measures for the CP sample. Psychometric measures of depression and anxiety were also collected.

### 2.1. Participants

CP patients were recruited in Italy from the Pain Center of the Humanitas San Pio X Clinic, Milan, also with the assistance of the Association of Rheumatoid Patients, Lombardy (ALOMAR). CP assessment and diagnosis were performed by the neurologist and pain specialist employed at the Pain Center. Given the clinical prevalence at the Center, Study 1 compared patients with: chronic primary pain (PP); chronic secondary musculoskeletal pain (SMP); and chronic neuropathic pain (NP); versus pain-free controls (PF). An a priori power calculation (f = 0.4, α err prob. = 0.05, power = 0.80, number of groups = 4, Critical F = 2.73) based upon previous literature [9,21], indicated a required total sample size of 76. A further a priori calculation for the regression analysis (f = 0.3, α err prob. = 0.05, power = 0.80, number of predictors = 6, Critical F = 2.30) recommended a sample size of 53 for the CP participants. The final sample was, therefore, composed of 80 participants comprising: 60 CP patients (47 women; Age M = 58.15, SD = 13.46; BMI M = 23.86, SD = 4.05); and 20 healthy participants as the control group. Healthy participants were matched for age and sex [16 women; Age M = 54.00, SD = 20.69; BMI M = 24.11, SD = 4.51] recruited via snowball consecutive sampling through university advertisements. All patients underwent a detailed general and neurological examination to obtain an accurate pain history. Eligible CP participants were adults, with normal cognitive function and language skills (as assessed by the neurologist and pain specialist at the Pain Center), with an active diagnosis of CP, who had experienced daily pain (>=4/10) for at least 3 months [3]. Patients were asked to continue their prescribed medication; avoid pain rescue medications in the 8 h before the experiment; and avoid nicotine and caffeine in the 2 h before the experiment. Participants in the control group underwent a detailed anamnestic interview (i.e., a specialized, comprehensive interview conducted by a clinician, to collect information on the patient’s or participant’s medical history and health status and the impact of this on his/her life) by a researcher specialized in psychopathological assessment. Exclusion criteria for the control group were: the presence of pain (acute or chronic); current diagnoses of psychological or physical disorders; disorders of sensory signs and symptoms such as hypoaesthesia, paraesthesia, allodynia or hyperalgesia; cardiovascular conditions; and pregnancy or lactation. The control participants were asked to avoid pharmacological medication in the 12 h before the experiment and nicotine and caffeine in the 2 h before the experiment. Compliance was confirmed during the anamnestic interview, along with clinical history, medications etc. All participants gave written informed consent, in accordance with the Declaration of Helsinki (2008). The protocol was approved by the Ethics Committee of Catholic University of Sacred Heart of Milan and by the Ethics Committee of Humanitas San Pio X Clinic.

### 2.2. Chronic Pain Assessment and Classification

Following evaluation, the CP patients were divided into three groups, according to the classification of their chronic pain for ICD-11 [3]: chronic primary pain (*N* = 23); chronic secondary musculoskeletal pain (*N* = 19); and chronic neuropathic pain (*N* = 18). Chronic primary pain (PP) is defined as pain in one or more anatomical regions that: persists or recurs for longer than 3 months; it is associated with significant emotional distress and/or significant functional disability; and where the symptoms are not better accounted for by another diagnosis [1]. Following the IASP guidelines [1], patients with musculoskeletal conditions for which the causes were incompletely understood, such as nonspecific back pain or chronic widespread pain, were grouped under chronic primary pain. Chronic secondary musculoskeletal pain (SMP) is a condition that arises either from an underlying disease “related to chronic nociception originating in the vertebral column, joints, bones, muscles, tendons and related soft tissues, from local or systemic aetiologies (and) also related to deep somatic lesions” or to diseases of the nervous system that may cause musculoskeletal pain [2].

Chronic neuropathic pain (NP) is defined as pain caused by a lesion or disease of the somatosensory nervous system [45,46]. Evaluation of the patient according to the widely adopted grading system [47] was undertaken if the patient’s history suggested that pain could be related to a neurological lesion or disease [48].

### 2.3. Experimental Procedure

After giving informed consent, all participants took part in a brief anamnestic interview with a researcher specialized in psychopathological and personality assessment. CP patients were assessed for pain condition (BPI-SF). All participants then completed psychological assessment for depression (BDI-II) and state anxiety (STAI). Finally, interoceptive accuracy (IAcc), interoceptive confidence (IAconf) and interoceptive sensibility (IAs) were measured, in all participants.

### 2.4. Interoceptive Accuracy

Interoceptive accuracy (IAcc) assesses the participant’s ability to accurately perceive inner bodily sensations. Although measures of interoceptive accuracy have recently been piloted in different modalities [49,50], cardiac interoceptive accuracy measured by heartbeat counting [18] has been by far the most commonly used measure of interoceptive accuracy in the literature on: the role of interoception in emotional intensity [51]; emotion regulation [52]; cortical signatures of interoception [53,54]; and the degree of activation of cortical areas connected to the interoceptive matrix [55]. Interestingly, low IAcc (measured by heartbeat counting) has been linked to a variety of mental disorders [11] and also to CP conditions [9,20]. Heartbeat counting was therefore employed in Study 1 for ease of comparison with this literature. In the heartbeat counting task, participants are required to silently count their heartbeats, for short intervals marked by two audio cues, by focusing only on inner bodily sensations, without taking their pulse. No feedback is given. The reported number of heartbeat is compared to the actual recorded number of heartbeats (measured with ECG) and the IAcc score (for n counting trials) is calculated as: 1/n∑(1–(|recorded heartbeats – counted heartbeats|) / recorded heartbeats). Scores vary between 0 and 1, with lower values indicating poorer performance. In Study 1, participants sat quietly in a comfortable chair and were connected to a portable ECG unit sampling at 1000 Hz with 3 Ag/AgCl electrodes. The trial intervals were 25 s, 35 s and 45 s. We used a ”strict instruction” to report only the heartbeats they actually felt, without guessing or estimating, as this minimises the confound that participants might use time estimation, or prior beliefs about their heart rate [56].

### 2.5. Interoceptive Confidence

Interoceptive confidence (IAconf) is a construct originally introduced by Garfinkel, Seth [19], whereby participants report how confident they are about their response on each of the heartbeat counting trials. Responses are given on a Visual Analogue Scale (VAS), Study 1 ranging from 0 (Not Confident at All) to 100 (Fully Confident). Garfinkel et al. (2015) found that mean confidence scores correlated with interoceptive cardiac accuracy, as measured by heartbeat counting [19] and also used confidence scores to calculate a metacognitive index, in terms of the within-participant Pearson correlation for each individual between confidence and accuracy across their heartbeat counting trials [19]. However, to be reliable this metacognitive measure requires a large number of trials. Therefore, the mean VAS confidence scores we collected were used a measure of interoceptive confidence, as in other literature, where interoceptive confidence has been shown to be low in clinical populations [57,58,59]. It has also been suggested, in predictive coding terms, that confidence may index the ‘precision’ of interoceptive sensations [60].

### 2.6. Interoceptive Sensibility

Study 1 employed the Multidimensional Assessment of Interoceptive Awareness (MAIA) [61] to measure interoceptive sensibility (IAs), which refers to the participant’s self-reported cognitive beliefs about their ability to perceive and interpret their bodily perceptions. This 32-item questionnaire has 8 subscales, which measure the participant’s: awareness of uncomfortable, comfortable and neutral body sensations (NO); tendency not to ignore or distract themselves from sensations of pain or discomfort (ND); tendency not to worry or experience emotional distress with sensations of pain or discomfort (NW); ability to sustain and control attention to bodily sensations (AR); awareness of the connection between bodily sensations and emotional states (EA); ability to regulate distress by attention to bodily sensations (SR); ability to actively listen to their body for insight (BL); and experience of their body as safe and trustworthy (TR). All responses are given on 6-point Likert scale.

### 2.7. Pain and Mood Measures

Pain condition was assessed through the Brief Pain Inventory Short Form (BPI-SF) [62], a robust and reliable instrument that specifically assesses clinically significant pain (which differs from common daily pain) experienced within the past 24 h.

Two composite indexes are calculated - a Pain Severity Score (PSS) for the individual’s pain level and a Pain Interference Score (PIS) for how much the pain interferes with daily life. Both have a maximum of 10.

Depressive mood was measured through the Beck Depression Inventory (BDI-II) [63], a well-validated and widely used 21-item questionnaire that discriminates different levels of depression. Scores under 13 indicate normal mood, while scores above 14 differentiate mild, moderate and severe depressive states [64,65].

In our analyses we always used total BDI-II scores but, for completeness, we report the two factors that index somatic and cognitive factors of depression [64,65]. Anxiety was measured with the well-validated 40-item State-Trait Anxiety Inventory (STAI) [66], which is regularly used with clinical and non-clinical participants. Scores above 40 indicate clinical levels of anxiety both in trait and state conditions. Given that pain is always a state measure and that our interoceptive measures were also collected as state variables, we used state anxiety (STAI_S) throughout our analyses.

### 2.8. Statistical Analyses

Given the non-Gaussian distribution of the main variables of interest and the unequal size of the groups (due to the random consecutive sampling), non-parametric tests were employed. Initially, we checked for any significant differences in age, sex and BMI between the groups. A series of Kruskal-Wallis tests were then used to identify significant differences between groups for: depression (BDI-II); state anxiety (STAI_S); IAcc; IAconf; and IAs (for each MAIA subscale). Post-hoc tests were performed using Dwass-Fligner, with Bonferroni correction for multiple comparisons. Similar analyses were conducted between the three CP subgroups (PP, SMP, NP) for: pain levels (PainNRS); pain duration (PainYRS); BPI Pain Severity Score (PSS); and BPI Pain Interference Score (PIS).

A multiple regression analysis was then performed for the whole CP sample. Previous literature suggested there would be a positive relationship between IAcc and pain [42] and between IAcc and IAconf [19,67] and a negative relation between IAcc, anxiety and depression [43,44]. Additionally, depression and anxiety are inter-related and both are linked to pain [4,5]. The analysis was therefore conducted with PainNRS as dependent variable and with predictors comprising: IAcc; IAconf; BDI; STAI_S; the interaction IAcc × IAconf; and the interaction BDI × STAI_S. All variables were centred before entering the regression analysis and the two interaction terms were calculated using standardized z-scores. Following methodological recommendations [68], all the low-level terms were left in the regression. Residual plots were checked for heteroscedasticity and for normality for observed standardized and unstandardized residuals.

It was important to check for possible confounding effects of psychiatric comorbidities or medications on interoceptive variables. We therefore used a stratified analysis in the CP sample, as in previous studies on CP and interoception [20,21]. Thus, additional ANOVAs were computed (with non-parametric tests used where appropriate) to compare CP participants suffering or not suffering from depression or anxiety, and CP participants using or not using anxiolytics, antidepressants, opioids, non-opioid analgesics, and antiepileptic medications (e.g., gabapentin).

Kruskal–Wallis tests, post-hoc analyses, regression analysis and boxplots were conducted in R Studio Version 1.1.463, using the following packages: *ggplot*; *bda*; *gvlma*; *and ggstatsplot* [69].

## 3. Results Study 1

### 3.1. Sample Characteristics, Pain Measures, and Psychological Measures

Table 1 and Supplementary Material Table S1 show the specific pathologies of CP participants. Overall, CP participants reported moderate levels of: pain (PainNRS M = 4.25; SD = 2.77); pain severity (PSS M = 4.728; SD = 2.16); and pain interference (PIS M = 4.92; SD = 2.55); with an average duration of pathology of 10.70 years (SD = 7.59). There were significant differences in the intensity of pain (PainNRS) between CP subgroups (χ^2^(2) = 8.75, *p* = 0.013). Primary pain participants (M = 5.52; SD = 2.89) were the most compromised, with higher levels of pain compared to secondary musculoskeletal (M = 3.21; SD = 2.84; *p* = 0.037) and to neuropathic pain participants (M = 3.72; SD = 1.87; *p* = 0.037). There were no significant differences in pain duration [PainYRS] between CP subgroups (*p* = 0.160). Results by CP condition (PF, SMP, PP, NP), are shown in Table 2.

CP participants, taken as a whole [*N* = 60], exhibited low mean interoceptive accuracy [M = 0.36; SD = 0.32], and low interoceptive confidence [M = 33.38; SD = 29.05]. The CP sample reported high mean levels of state anxiety [STAI_S M = 40.27; SD = 13.09] and depression [BDI M = 17.70; SD = 11.40]. These mean scores are all above clinical cut-offs. Chi-square tests showed no statistically significant differences in gender [*p* = 0.88], age [*p* = 0.95] or BMI [*p* = 0.76] between CP patients and healthy participants.

Results, divided by CP condition [PF, SMP, PP, NP], are shown in Table 2. A full correlation matrix for all relevant variables is given in Table 3.

### 3.2. Interoceptive Accuracy

**Figure 1:**
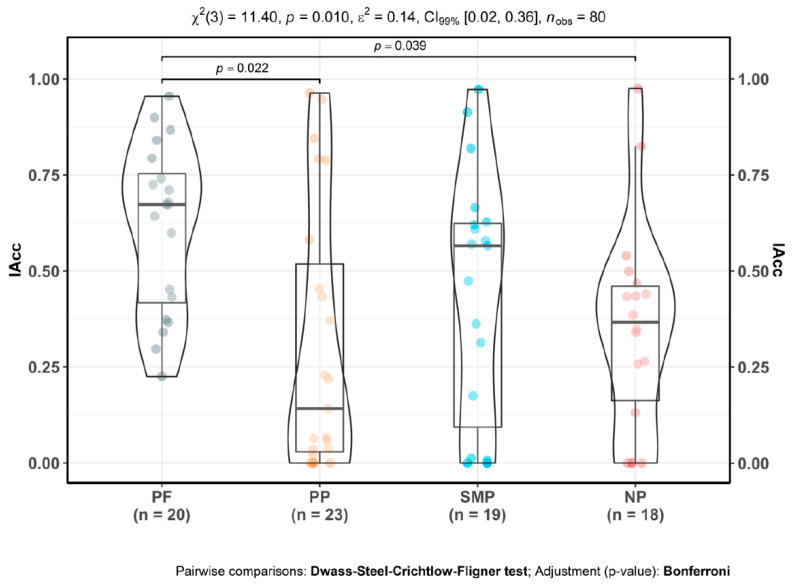
Interoceptive accuracy (IAcc) alterations in CP. PF: pain-free participants. PP: primary CP participants, SMP: secondary musculoskeletal CP participants, NP: neuropathic CP participants. Mean values: PF µ = 0.61, CI_95%_ (0.51, 0.71), PP µ = 0.31, CI_95%_ (0.16, 0.46), SMP µ = 0.44, CI_95%_ (0.29, 0.59), NP µ = 0.35, CI_95%_ (0.22, 0.48).

There were significant differences in interoceptive accuracy between groups [PF, SMP, PP, NP], as determined by Kruskal–Wallis test [χ^2^(3) = 11.40, *p* = 0.01]. Primary pain [*M* = 0.31; *SD* = 0.35; *p* = 0.02] and neuropathic pain participants [*M* = 0.35; *SD* = 0.27; *p* = 0.04] had significantly lower IAcc compared to pain-free controls [*M* = 0.61; *SD* = 0.22]. By contrast, secondary musculoskeletal pain participants [*M* = 0.44; *SD* = 0.32] showed no significant IAcc differences from controls [*p* = 0.21]. There were no significant differences between CP subgroups (Figure 1). Violin plots show the complete distribution of the data in the form of: scatterplot, where each dot represents a single data point; curve indicating the probability density of the distribution; and superimposed box plot, showing the median, quartiles and inter-quartile range.

### 3.3. Interoceptive Confidence

**Figure 2:**
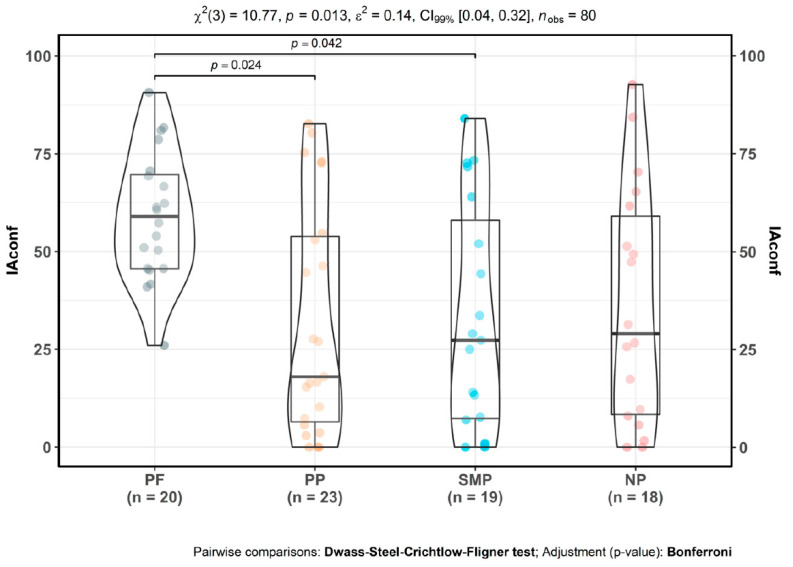
Interoceptive confidence (IAconf) alterations in CP. PF: pain-free participants. PP: primary CP participants, SMP: secondary musculoskeletal CP participants, NP: neuropathic CP participants. Mean values: PF µ = 59.05, CI_95%_ (51.36, 66.74), PP µ = 31.9, CI_95%_ (19.22, 44.58), SMP µ = 32.67, CI_95%_ (18.68, 44.66), NP µ = 36.02, CI_95%_ (21, 51.04).

There were significant differences in interoceptive confidence (Figure 2) between groups, as determined by Kruskal-Wallis test [χ^2^(3) = 10.72, *p* = 0.01]. Primary pain [*M* = 31.90; *SD* = 29.33; *p* = 0.02] and secondary musculoskeletal pain participants [*M* = 32.67; *SD* = 29.03; *p* = 0.04] were less confident about their interoceptive perception compared to controls [*M* = 59.05; *SD* = 16.43]. No significant differences were found for neuropathic pain [*M* = 36.02; *SD* = 30.21, *p* = 0.10]. There were no significant differences between CP subgroups. Moreover, in the CP sample, IAconf was negatively correlated with all the mood measures: BDI [−0.29, *p* = 0.02]; and STAI_S [−0.44, *p <* 0.001].

### 3.4. Interoceptive Sensibility

There were no significant differences between groups on any of the MAIA subscales [*p >* 0.05]. Scores and results for each subscale are reported in Table 2.

### 3.5. Depression and Anxiety

**Figure 3:**
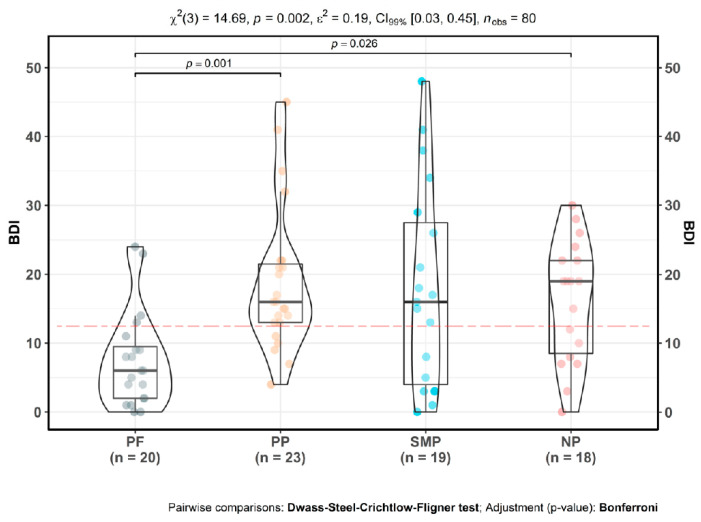
Depressive symptoms in CP. PF: pain-free participants. PP: primary CP participants, SMP: secondary musculoskeletal CP participants, NP: neuropathic CP participants. Mean values: PF µ = 7.5, CI_95%_ (4.29, 10.71), PP µ = 18.83, CI_95%_ (14.31, 23.35), SMP µ = 17.84, CI_95%_ (10.77, 24.91), NP µ = 16.11, CI_95%_ (11.73, 20.49). Red dotted line indicates clinical cut off for depressive symptoms, scores above 14 differentiate mild, moderate and severe depressive states [65,66]).

Overall, all the CP groups exhibited clinical levels of depressive symptoms, with mean BDI total scores above clinical cut-offs (Figure 3). However, there were significant differences in depression scores between groups, as determined by Kruskal–Wallis test [χ^2^(3) = 14.69, *p* = 0.002]. Closer investigation showed that both primary pain [*M* = 18.83; *SD* = 10.45; *p <* 0.001] and neuropathic pain participants [*M* = 16.11; *SD* = 8.79; *p* = 0.03] had significantly higher BDI total mean scores compared to pain-free controls [*M* = 7.50; *SD* = 6.84]. However, in secondary musculoskeletal pain, BDI mean total scores were not significantly higher [*M* = 17.84; *SD* = 14.67] than in pain-free participants [*p* = 0.12]. There were no significant differences between CP subgroups.

Likewise, all the CP groups exhibited high mean levels of state anxiety (STAI_S), close to or above clinical cut-offs.

**Figure 4:**
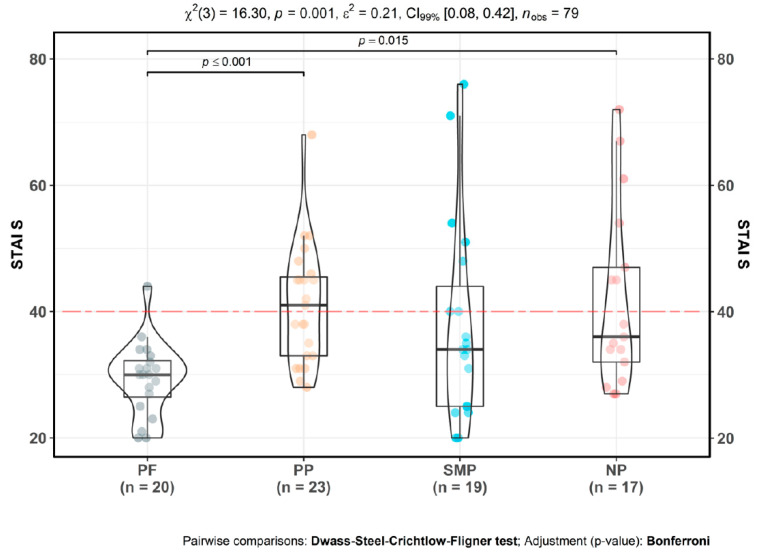
State anxiety in CP. PF: pain-free participants. PP: primary CP participants, SMP: secondary musculoskeletal CP participants, NP: neuropathic CP participants. Mean values: PF µ = 29.45, CI_95%_ (26.73, 32.17), PP µ = 41.04, CI_95%_ (36.92, 45.16), SMP µ = 37.95, CI_95%_ (30.28, 45.62), NP µ = 41.82, CI_95%_ (34.53, 49.11). Red dotted line indicates clinical cut off for state anxiety symptoms. Scores above 40 indicate clinical levels of anxiety [67].

There was a statistically significant difference (Figure 4) between groups [χ^2^(3) = 16.30, *p <* 0.001]. Primary pain [*M* = 41.04; *SD* = 9.52; *p* <= 0.001] and neuropathic pain participants [*M* = 41.82; *SD* = 14.18; *p* = 0.02] had significantly higher state anxiety compared to pain-free participants [*M* = 29.45; *SD* = 5.88]. Once again, secondary musculoskeletal pain participants were the exception [*M* = 37.95; *SD* = 15.92; *p* = 0.25] reporting subclinical anxiety levels that were not significantly different from pain-free participants. There were no significant differences in state anxiety between CP subtypes.

### 3.6. The Relationship between CP, Interoception and Mood

To further investigate the relationship between CP and interoception, a multiple regression analysis was conducted with PainNRS as dependent variable and IAcc, IAconf, BDI, STAI_S, the interaction term IAcc × IAconf and the interaction term BDI × STAI_S as predictors.

As hypothesised, taken together, these variables significantly predicted the intensity of CP participants’ pain [R = 0.66, R^2^ = 0.44, F(6, 52) = 6.77, *p* < 0.001, AIC = 268.01, BIC = 284.63] (missing values in at least one of the specified variables were addressed with listwise deletion).

Standardized Beta coefficients indicated that: IAcc [β = 0.35, *p* = 0.01]; BDI scores [β = 0.34, *p* = 0.02]; and STAI_S scores [β = 0.37, *p* = 0.02] positively predicted subject’s levels of pain, while IAconf [β = −0.287, *p* = 0.04] was a negative predictor. Both interaction terms were also significant IAcc × IAconf [β = 0.40, *p* <= 0.001], BDI × STAI_S [β = −0.24, *p* = 0.04].

For completeness, regression models with only interoceptive variables [R^2^ = 0.15, F(3, 56) = 3.24, *p* = 0.03, AIC = 291.73, BIC = 302.20] and mood variables [R^2^ = 0.22, F(3, 55) = 5.18, *p* = 0.003, AIC = 281.36, BIC = 291.74] were also tested and were both significant.

However, both of these models resulted in higher AIC and BIC scores and lower R^2^ scores compared to our full, hypothesis-based, model shown above.

### 3.7. A Check for Potential Confounding Factors of Medication and Comorbidity in CP Patients

To control for possible confounding effects on interoceptive measures of psychiatric comorbidities and medication in our CP patients (shown in Table 4), we used a stratified analysis in the CP sample [20,21]. Additional ANOVAs were computed (with non-parametric tests where appropriate). Results indicated that there were no significant differences in interoceptive measures between: patients suffering vs. not suffering from depression [IAcc *p* = 0.23, IAconf *p* = 0.44] (stratified analysis for anxiety disorders was not run due to the different size of the groups. As reported in Table 4, only four participants were suffering from anxiety disorders. A comparison of four participants vs 56 participants not suffering for anxiety disorders would have yield no statistical reliability). Similarly, no differences were found between patients taking vs. not taking: anxiolytic medication [IAcc *p* = 0.06, IAconf *p* = 0.80]; antidepressants [IAcc *p* = 0.10, IAconf *p* = 0.41]; opiates [IAcc *p* = 0.59, IAconf *p* = 0.15]; non-opiate analgesics [IAcc *p* = 0.58, IAconf *p* = 0.57]; and antiepileptic medications [IAcc *p* = 0.75, IAconf *p* = 0.10]. Together, these results indicate that psychiatric comorbidities and medication did not cause the differences in interoceptive measures we observed in the CP sample.

## 4. Materials and Methods Study 2

CP is a complex pathological condition that poorly responds to pain management treatments and therapies. Study 2 presents a novel potential interoceptive treatment for CP, using a non-invasive method to induce analgesia through interoceptive stimulation of the C-Tactile afferents in the skin. This study employed a single-blind, between-subject design, in primary, secondary musculoskeletal, and neuropathic CP patients, to test the ability of specific CT interoceptive stimulation to reduce pain symptom severity.

### 4.1. Participants

At the conclusion of Study 1, CP participants were requested to rate the pain connected to their chronic condition (Pain NRS measures were collected after the interoceptive tasks, thus allowing the subject to rest comfortably for approximately 20 min before taking this measure. This ensured that the reported pain was properly stabilized and was due to the chronic condition) on a Numeric Rating Scale (PainNRS) from 0 to 10. Those who reported active pain were invited to participate in Study 2.

Fifty-one participants were eligible. An a priori calculation [f = 0.22, α err prob. = 0.05, power = 0.80, number of groups = 2, number of measurement = 2, correlation among rep. measure = 0.5, non-sphericity correction = 1, Critical F = 4.072] based upon previous literature [35,36,37] indicated a required total sample size of 44 participants. Of the 51 potential CP participants, two were later excluded because they could not complete the procedure for medical reasons (subjects were not able to sit on the examination bed because the posture increased the pain). The final sample was composed of 49 CP patients [39 women; Age M = 57.92, SD = 14.48; BMI M = 23.90, SD = 4.33; PainPre_NRS M = 4.89, SD = 2.24; BDI M = 18.06, SD = 11.05; STAI_S M = 42.17, SD = 13.23]. There were 19 PP participants, 13 SMP and 17 NP participants. Details of psychometric scores and demographic data for the experimental and the control group are provided in Supplementary Figure S1.

### 4.2. Experimental Design and Procedure

Study 2 followed a single-blind, between-subjects design. Using a computer-generated, block randomization sequence (R psych library, *block.random* function), the CP participants were randomly assigned to the experimental condition [*N* = 24] in which they received interoceptive CT stimulation, or to the control condition [*N* = 25] where they received control stimulation with non-interoceptive tactile pressure. As a cover story, all participants were told that they would receive a series of non-painful (neutral) tactile stimuli and that their task was to estimate their time duration in seconds.

Experimental procedures for interoceptive touch often collect pleasantness ratings for the stimulation. However, we refrained, in order to avoid compromising the blinding of the condition, given that expectations and context are known to modulate pain perception [70]. We were therefore not able to explore the secondary research question of the processing of pleasant tactile sensations in CP, which is nonetheless a topic already explored within the literature [71].

### 4.3. Tactile Stimulation

Interoceptive touch has been defined as a secondary touch system [26,27,29] and it is activated only within specific parameters, namely with a moving stimulus at 3 cm/s and with a force of <40 mN and preferably = 2.5 mN [29,30,72,73]. Interoceptive tactile afferents, thus, selectively respond to low-velocity, low-force stimulation [24,72,74] but not to mechanical vibrational waves, high velocities or indentation force stimuli [24,29,31]. There is microneurographic evidence that describes this pattern of activation and demonstrates that low-velocity, low-force stimulation activates the C-Tactile small unmyelinated fibers that directly relay information to the insular cortex and to the interoceptive cortical network [24,28,74,75,76,77], rather than fast myelinated somatosensory fibers [30,78]. Moreover, it has been shown that, if these specific low-velocity, low-force parameters are met, the stimulation in primarily processed by the left insula [28], rather than by the somatosensory cortex. The defining characteristic of interoceptive touch is, therefore, the force and velocity of the stimulation. By contrast, our control condition (i.e., pressure at 100 mN) does not activate the interoceptive tactile system, which does not respond to high force indentation stimuli [24,29,31]. For example, microneurographic evidence with von Frey filaments shows that no activation of C-Tactile afferents occurs above 40 mN [29]. Moreover, pressure in the same range that we employed, has previously been used as a control condition [30,32,78] in several studies on interoceptive touch, precisely because it activates a different set of receptors, that are processed in the somatosensory and not the interoceptive cortex.

**Figure 5:**
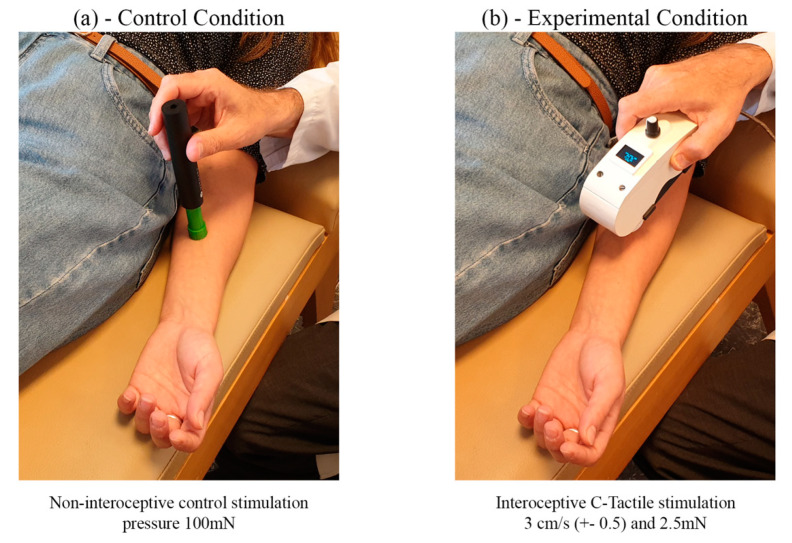
Stimulation procedure. (**a**) Control condition with pressure stimulation at 100 mN (**b**) experimental condition with optimal C-Tactile stimulation at 3 cm/s and with a force of 2.5 mN.

Therefore, following the procedure in Di Lernia, Serino [67] and Di Lernia, Cipresso [41], experimental and control conditions used these two essentially different forms of tactile stimulation. For the experimental condition this was interoceptive CT stimulation on the left volar forearm [24], provided by a specially developed instrument [41] (Figure 5b) that delivers a pattern of circular stimulation at 3 cm/s and 2.5 mN with a linear component of 0.5 cm/s, moving from elbow to wrist and back. This body site was chosen following microneurographic evidence in the literature confirming the presence of CT afferents in the volar forearm of healthy participants [79]. In the control condition in Study 2, participants received non-interoceptive stimulation applied to the same site with similar time parameters, moving from elbow to wrist and back along the left volar forearm in the same fashion as in the experimental condition. This non-painful, non-interoceptive pressure stimulation was delivered using a pre-calibrated cylinder with smooth edges (Figure 5a). Pressure was pre-determined by the weight of the cylinder on the skin (100 mN) following literature recommendations [32,78,80]. As described above, this provides an optimal control condition because, crucially, non-painful pressure stimuli are not processed by the interoceptive C-Tactile afferent system [32,78] In both conditions, tactile stimulation was delivered for 6 blocks, each comprising 6 short periods of stimulation of 8 s, 10 s, 12 s, 14 s, 16 s, and 18 s, presented in random order, with pauses of 6 s after every period of stimulation. The entire duration of the stimulation, in each condition, was approximately 11 min. Pain measures were collected immediately before and after the stimulation, with a Numeric Rating Scale (PainNRS) ranging from 0 (no pain) to 10 (the worst pain imaginable).

### 4.4. Statistical Analyses

To evaluate the effect of the interoceptive stimulation on CP, we first fitted a Linear Mixed Model (LMM) (estimated using REML and nloptwrap optimizer) to predict Pain, with Time (factors: PainPre_NRS and PainPost_NRS) and Condition (factors: Control and Interoceptive Stimulation), according to the following formula = Pain ~ Time * Condition. The model included the individual participants as random effects (formula = ~1 | ID).

To check for the possible effect of the clinical cluster on the results, we then fitted a second Linear Mixed Model (estimated using REML and nloptwrap optimizer) again to predict Pain, but now including Time (factors: PainPre_NRS and PainPost_NRS), Condition (factors: Control and Interoceptive Stimulation), and Clinical Cluster (PP, SMP, and NP), according to the following formula = Pain ~ Time * Condition * Clinical Cluster. This model also entered the individual participants as random effects (formula = ~1 | ID).

Assumptions for both the models were satisfied. Analysis of variance (ANOVA) on Linear Mixed Model parameters was performed with Kenward-Roger approximation for degrees of freedom [81]. Post-hoc comparisons with Bonferroni correction were performed with *emmeans* and are reported with estimated marginal means (EEM) and standard error (SE). The Linear Mixed Models were run in R Studio Version 1.1.463 with the following packages *Lme4* [82] with restricted maximum likelihood [83], *lmerTest* [84], *emmeans* [85].

We modeled differences between the control and experimental groups regarding pain levels at baseline in our post hoc analyses. This provides the best estimate of possible differences because it corrects for multiple comparisons. In doing so, we follow the recommended statistical guidelines, accounting for these differences directly in our statistical models, rather than checking them at the baseline with e.g., t-tests [86]. Furthermore, to investigate the effect of possible confounding variables, we controlled for age, BMI, IAcc, IAconf, BDI, and STAI_S in the models.

In Supplementary Material Figure S1 we also show traditional repeated measures (RM) ANOVAs, with factor Time (PainPre_NRS and PainPost_NRS), factor group (Control and Experimental) and factor Clinical Cluster (PP, SMP, NP), controlling for all relevant interoceptive, mood, and demographic covariates. Results from RM ANOVAs confirm those of the LMMs.

## 5. Results Study 2

### Pain Reduction after Interoceptive Tactile Stimulation

Results of the LMMs indicated that there was a significant reduction in perceived pain following the interoceptive stimulation. The total explanatory power of the first Linear Mixed Model was substantial (conditional R2 = 0.90). The part related to the fixed effects alone (marginal R2) was 0.15. The model’s intercept, corresponding to Pain = 0, Time = PainPre_NRS, Condition = Control and ID = 1, was at 5.52 (SE = 0.45, 95% CI [4.64, 6.40], std. intercept = 0.35, *p* < 0.001). Analysis of variance (ANOVA) on this Linear Mixed Model’s parameters indicated a significant main effect of Time [F (1, 47) = 8.6796, *p* = 0.005, η_p_^2^ = 0.148, Cohen’s f = 0.417] and a significant main effect of Condition [F (1, 47) = 8.1159, *p* = 0.006, η_p_^2^ = 0.140, Cohen’s f = 0.403]. More importantly, the interaction effect of Time × Condition was significant [F (1,47) = 10.2577, *p* = 0.002, η_p_^2^ = 0.170, Cohen’s f = 0.453]. Post hoc analyses indicated that the Experimental group who received the interoceptive stimulation reported a significant reduction of pain [EMM estimate = −0.958, SE 0.223, p_b_ < 0.001] between pain before the stimulation [PainPre_NRS EMM = 4.25, SE = 0.457] and pain after the stimulation [PainPost_NRS EMM = 3.29, SE = 0.457]. No significant differences in perceived pain were found in the Control group following the stimulation [EMM estimate = 0.040, SE = 0.218, p_b_ > 0.05]. Moreover, no significant differences were found in pain at baseline between the Control and Experimental groups [EMM estimate = 1.270, SE = 0.640, p_b_ = 0.315].

**Figure 6:**
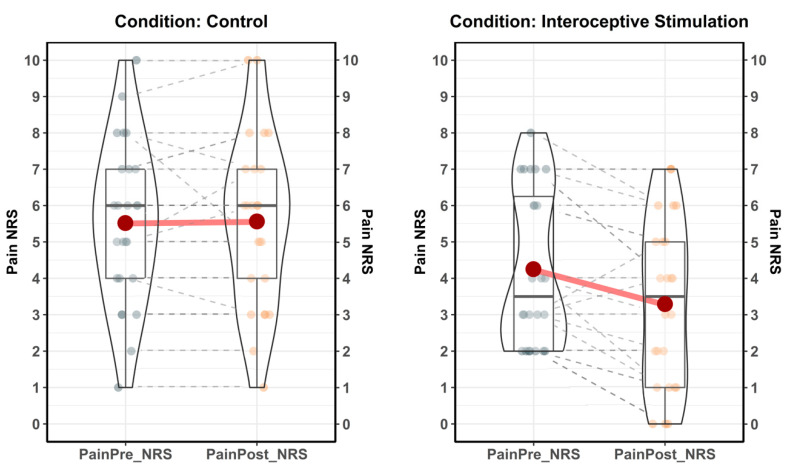
Interoceptive stimulation reduces Chronic Pain. In the control condition, CP patients (*N* = 25) received control tactile pressure stimulation at 100 mN. In the experimental condition, CP patients (*N* = 24) received interoceptive tactile stimulation at 3 cm/s with a force of 2.5 mN. Red dots represent mean values. Control condition, PainPre_NRS µ = 5.52, CI_95%_ (4.6, 6.44), PainPost_NRS µ = 5.56, CI_95%_ (4.6, 6.52). Interoceptive stimulation condition, PainPre_NRS µ = 4.25, CI_95%_ (3.35, 5.15), PainPost_NRS µ = 3.29, CI_95%_ (2.33, 4.25).

In summary, results (Figure 6) indicate that those CP patients who received interoceptive C-Tactile stimulation reported a pain reduction of 22.58%, on average, compared to baseline values [PainPre_NRS, M = 4.25, SD = 2.13; PainPost_NRS, M = 3.29, SD = 2.27; ∆Pain, M = −0.958, SD = 1.268], after approximately 11 min of stimulation. Conversely, CP patients who received control (pressure) stimulation did not exhibit any pain reduction compared to baseline [PainPre_NRS, M = 5.52, SD = 2.22; PainPost_NRS, M = 5.56, SD = 2.32; ∆Pain, M = 0.040, SD = 0.889].

The results of this first model remained significant after controlling for all the relevant cofounding variables, testing for possible three-way interaction with factors Time and Condition. None of the relevant confounding variables significantly interacted: demographics [Age *p* = 0.87, BMI *p* = 0.29, pain duration in years *p* = 0.76], mood [BDI *p*= 0.41, STAI_S *p* = 0.91]. Moreover, neither of the interoceptive variables [IAc *p* = 0.96, IAw *p* = 0.68] had any significant effect on pain reduction.

Results from the second Linear Mixed Model confirmed the results of the first model and indicated that there was no significant effect of the Clinical Cluster, implying that the CT stimulation was effective in reducing pain independently from of the pathological condition. Specifically, Analysis of variance (ANOVA) on this Linear Mixed Model’s parameters indicated a significant main effect of Time [F (1, 47) = 7.3318, *p* = 0.009, η_p_^2^ = 0.136, Cohen’s f = 0.397] and a significant main effect of Condition [F (1, 47) = 10.7661, *p* = 0.002, η_p_^2^ = 0.188, Cohen’s f = 0.481]. There was a significant main effect of Clinical Cluster [F (1, 47) = 5.2655, *p* = 0.009, η_p_^2^ = 0.185, Cohen’s f = 0.476], indicating a significant difference in pain levels between clinical clusters, and specifically that PP patients reported more pain than NP patients [EMM estimate = 2.067, SE = 0.658, p_b_ = 0.009], as also found in Study 1. However, there were no significant interaction of Clinical Cluster × Condition [*p* = 0.22], Clinical Cluster × Time [*p* = 0.65], or Clinical Cluster × Time × Condition [*p* = 0.72]. More importantly, the interaction effect of Time × Condition was significant [F(1,47) = 9.6884, *p* = 0.003, ηp2 = 0.172, Cohen’s f = 0.456] and post-hoc analyses confirmed the analgesic effect of the interoceptive stimulation [EMM estimate = 0.958, SE = 0.223, t.ratio = 4.304 *p* < 0.001]. However, the second LMM was slightly underpowered, therefore conclusions must be draw with caution.

Nonetheless, taken together, these results confirm the analgesic effect of the CT stimulation and indicate that the interoceptive tactile analgesia was effective, independently of the pathological condition.

## 6. Discussion

Given the fundamental relevance of interoception to Chronic Pain (CP), the purpose of the two studies presented here was firstly to measure different facets of interoceptive perception across several chronic pathological pain conditions, compared with healthy pain-free controls. In Study 1, lower interoceptive accuracy and interoceptive confidence were found across CP patients, taken as a whole, compared to pain-free controls. In Study 2, with CP patients, we tested the analgesic effects of an innovative method of CT interoceptive stimulation, compared with a control of non-interoceptive stimulation. CT interoceptive stimulation significantly reduced the severity of reported pain in primary, secondary musculoskeletal and neuropathic CP patients, indicating that this method has the potential to provide a valuable transdiagnostic complementary analgesic treatment.

Study 1 explored the relationship between pain, cardiac interoceptive accuracy, interoceptive confidence, self-reported interoceptive sensibility and mood measures for depression and anxiety, as these interact with both CP and interoception.

Results revealed a pattern not previously reported in the literature. Overall, compared to healthy controls, CP participants were less able to accurately perceive inner bodily sensations and overall, they similarly had lower confidence in these perceptions. However, no significant differences were found between CP and pain-free participants in their self-reported beliefs about how well they thought that they could perceive their inner bodily sensations (i.e., their interoceptive sensibility measured by the MAIA questionnaire). This suggests that CP patients, in general, are unaware that the accuracy of their perception and/or their confidence in their interoceptive sensations is actually impaired, compared to healthy controls. This pattern was seen across all three CP subgroups, compared to pain-free controls suggesting that CP involves disrupted signaling (interoceptive accuracy) or integration (interoceptive confidence) of body to brain, with consequently poorer ability to process non-pathological bodily sensations. However, CP patients are unconscious of this disruption (have no differences in interoceptive sensibility).

No significant differences in interoceptive measures were found between any of the CP subgroups, although primary and neuropathic pain participants appeared to be the most compromised across interoceptive measures, confirming previous findings [21,87]. By contrast, secondary musculoskeletal pain participants were characterized by reduced interoceptive confidence only, without significantly lower interoceptive accuracy or sensibility than controls, which has also previously been reported [88]. While none of the CP groups were significantly different in any interoceptive or mood measure, when compared to each other, the interoceptive pattern in the CP clinical cluster deviated from healthy controls in two specific points. The NP group exhibited no significant deficit in interoceptive confidence, while the SMP group showed no deficit in interoceptive accuracy. We believe that the difference in NP is driven by the statistical distribution of the values in our NP group, which has very large confidence intervals. By contrast, the absence of deficits in interoceptive accuracy in the SMP cluster may have a different explanation, as Ribera D’Alcalà and colleagues found a pattern similar to ours [88]. Those authors suggest that people with SMP conditions are often subjected to osteo-manipulative treatments and physical rehabilitation. Importantly, these treatments involve touch and manipulation of the body. This may enhance interoceptive awareness and body perception and could also drive a less compromised condition (i.e., SMP had lower levels of pain compared to the other patient clusters).

Importantly, when we explored the impact of these variables in Study 1, using multiple regression, pain intensity in CP participants (regardless of diagnosis) was positively predicted by interoceptive accuracy, anxiety and depression, but negatively predicted by interoceptive confidence. The apparent paradox of low mean IAcc in CP but positive correlation of IAcc with pain severity suggests that interoceptive accuracy, which indexes a person’s ability to correctly perceive their body, is associated with enhanced perception of all patients’ bodily sensations, including pain. Such an explanation in CP participants is in keeping with previous literature on healthy individuals, where high interoceptive accuracy has been shown to correlate positively with both decreased tolerance of acute pain and enhanced perception of acute pain [42] and paradoxical pain experiences [89]. Additionally, deficits in IAc have previously been found in various CP populations [21,87]. However, our study is the first to examine these two trends together. Our hypothesis is that the presence of chronic pain interferes with the perception of other bodily-related stimuli (e.g., heartbeats, as measured interoceptive accuracy). Thus, CP patients, taken as a whole, have relatively low IAc compared to healthy controls. Speculatively, CP patients may suppress their interoceptive accuracy as a means to avoid pain. However, there is a paradox that, within CP, the ability to perceive bodily sensations (which is what IAc indexes) inevitably enhances the perception of pain, via somatosensory amplification mechanism and bodily sensation hypervigilance [90], where extreme attention to bodily inputs contribute to pain perception and symptoms severity. Thus, within CP, higher IAc is accompanied by greater pain (as in healthy people).

Somewhat surprisingly, however, in CP patients interoceptive confidence negatively predicted pain severity as well as being associated with lower anxiety and depression (indicated by negative correlations between interoceptive confidence, depression and anxiety). This implies that confidence in one’s bodily perceptions may play a protective role against pain severity in CP. For example, if high interoceptive accuracy compromises functioning due to somatosensory amplification, as in paradoxical pain and in fear avoidance models [90,91], then interoceptive confidence might have the effect of correcting evaluations of those paradoxical and hypervigilant perceptions and down-regulating them.

The pattern we observed in our regression analysis indicates that intensity of pain in CP does not simply result from facets of interoception but emerges comorbidly with depression and anxiety, which are both known to be important factors in pain perception [4,5,92,93]. Both depression and anxiety have a direct impact on insula activity and the processing of interoceptive inputs, at the sympathetic and parasympathetic level. Specifically, recent neurophysiological evidence indicates that depression can alter connectivity, morphology, and functionality of the insular cortex [43,44,94,95,96,97,98,99]. By contrast, anxiety shifts the functional activity of the insular cortex [44,94,100] toward hyperactivation and towards enhancement of sympathetic processing of unpleasant stimuli [101]. Thus, our results imply a complex set of relationships between pain, interoception and mood, in chronic conditions, which merits further research with larger samples to examine the interactions. It may be, for example, that interoceptive confidence in inner bodily sensations can moderate alterations in mood, not only by reducing symptom severity but by helping patients to better manage the somatic aspects and emotional processing arising from their chronic condition, as preliminary correlational evidence here and in other clinical populations has suggested [59].

In Study 2 we tested CT interoceptive stimulation as a means to reduce pain severity in CP. To the best of our knowledge, interoceptive tactile stimulation has never previously been applied to modulate chronic pain in primary, musculoskeletal, and neuropathic conditions. Notwithstanding, we found that such stimulation, reduced pain intensity by an average of 23% in CP participants, after only 11 min of stimulation, demonstrating the potential of this innovative treatment, as a non-invasive complement to pain management in a variety of CP conditions, without pharmacological interference or side effects.

Although the mechanisms of interoceptive pain analgesia are yet to be fully elucidated, several hypotheses have been put forward. Recent evidence from animal models indicates that CT stimulation suppresses pain through a modulatory inhibitory effect in the dorsal horn, with a concomitant release of protein TAFA4 that has analgesic effects [38,39]. Similar results have been found for acute experimentally-induced thermal pain in humans, where C-Touch modulates thermal pain intensity in healthy participants [34,35]. Moreover, as demonstrated in previous studies [31,41], interoceptive CT stimulation can enhance heart rate variability, specifically in the high-frequency band, implying it has a direct effect upon the parasympathetic system, with a possible concomitant reduction of sympathetic, pain-related, activation. Likewise, there is evidence that interoceptive touch may mediate the µ-opioids system response [40] which is a promising target for CP treatment [102]. Possibly, CT stimulation may also mediate oxytocin release [33] and numerous studies have demonstrated that oxytocin has a direct effect upon several domains of pain perception - modulating pain intensity, anxiety, and depressive symptoms [103,104]. Overall, the analgesic effects of interoceptive stimulation are unlikely to rely on a single mechanism but are most probably fostered by a synergy of processes at autonomic, endocrine and cortical levels. Interestingly but unsurprisingly, when added as covariates, neither mood nor interoceptive variables were linked to the analgesic effect of the direct stimulation of CT afferents, implying that CT pain suppression mechanisms are independent of behavioural interoceptive perceptions and self-reported mood, as indicated by previous evidence also on animal models and whole-cell neurons direct recordings [38,39]. In conclusion, the results of our two Studies indicate the exciting possibility of developing new trans-diagnostic interoceptive treatments that can help to manage pain in chronic conditions, independently of the originating pathology – as our consistent results across three different CP aetiologies indicate. These kinds of treatment rely upon the concept of interoceptive technologies – namely a brand-new technological field that uses advanced solutions to enhanced and modulate the interoceptive system through different means, e.g., sounds and vibrational waves (Sonoception) [105] but also virtual reality [106], other than interoceptive touch [41,107].

### Limitations

Several limitations must be considered in the interpretation of our results. In Study 1: Critics of heartbeat counting [108] suggest that people either count at some familiar ”prior” rate or use knowledge of their own heart rates and time estimation strategies. To mitigate this, we utilized a strict instruction in the heartbeat counting task (“count only those beats you feel and do not guess”) as recommended by Desmedt, Corneille [57] to increase the reliability of results. Moreover, heartbeat counting is widely used in CP research generally [9] as well as in recent studies [20], which allows comparability of our results with other CP literature.

A limitation of Study 2 involves the control condition. Previous studies on interoceptive touch have utilized different velocities [75] or vibrational stimuli [31] as controls. This was impossible with CP participants because both rapid tactile stimulation and vibration can activate Aβ fibres which are connected to mechanical allodynia in several CP conditions and could elicit pain [109,110,111]. The current design followed Manzotti, Cerritelli [32] in using static pressure, which has no analgesic or painful effects.

Finally, the experimenters were not blind to the condition because interoceptive touch can only be administered with our specialized device. For a true double-blind methodology, the device would have to be able to provide both the CT and the control stimulation, without the experimenter being aware of which condition was being applied. This cannot yet be achieved technically.

## 7. Conclusions

Chronic pain (CP) is a complex condition that affects over 500 million people worldwide, with enormous costs to society and massive impact on patients’ quality of life [112,113,114]. Although the literature has suggested interoceptive deficits in CP conditions, no previous study has compared several different chronic pain syndromes against core facets of interoception.

Results from Study 1 identified low interoceptive accuracy and confidence across CP conditions. Moreover, interoceptive and mood variables predicted pain severity across the whole CP sample, with interoceptive accuracy positively predicting and interoceptive confidence negatively predicting pain severity.

In Study 2 we tested interoceptive tactile treatment in CP. With only 11 min of stimulation, this treatment reduced pain severity (by an average of 23%) compared to a control condition of non-interoceptive touch. As we note in the Introduction and in the Discussion, the literature suggests that interoceptive touch modulates the u-opioid and oxytocin responses, TAFA4 expression and HRV autonomic balance. These specific biological and autonomic markers have a blood half-life/effect ranging from several minutes (oxytocin, HRV) to several hours (TAFA4, and u-opioids) [38]. We anticipate that future studies will show that the biological response curve for interoceptive touch falls within the same time windows.

In conclusion, our study provides important evidence that treatments based upon interoceptive tactile stimulation can be an effective complementary tool in pain management. Future studies, with larger samples, will probe the temporal limits of the analgesic effect and the effects of regular application.

## Figures and Tables

**Table 1:** CP pathologies and cluster classification.

Diagnosis	Cluster Assignment		
	*Chronic Primary Pain*	*Chronic Secondary Musculoskeletal Pain*	*Chronic Neuropathic Pain*
Chronic primary pelvic pain	2		
Chronic tension-type headache	1		
Fibromyalgia	20		
Arthritis		8	
Osteoarthrosis		4	
Traumatic Rib Injury		1	
Low Back Pain / Spondylosis		5	
Paget’s disease		1	
*Central Neuropathic Pain*			
Syringomyelia			DNP: 2
Spinal cord injury			DNP: 1
*Peripheral Neuropathic Pain*			
Peripheral nerve injury			DNP: 6, PRNP: 3
Polyneuropathy			DNP: 3
Painful cervical radiculopathy			DNP: 2
Postherpetic neuralgia			DNP: 1
Total(N)	23	19	18

PNP: possible neuropathic pain, PRNP: probable neuropathic pain, DNP: definite neuropathic pain. According to the grading system of neuropathic pain [47].

**Table 2:** Sample characteristics and principal variables of interest.

		Healthy				Chronic Pain												
		*Pain-Free (N = 20)*				*Primary Pain (N = 23)*				*Secondary Musculoskeletal Pain (N = 19)*				*Neuropathic Pain (N = 18)*				
Demo		*Mean*	*SD*	*Min*	*Max*	*Mean*	*SD*	*Min*	*Max*	*Mean*	*SD*	*Min*	*Max*	*Mean*	*SD*	*Min*	*Max*	*p*
	Age	54.0	20.7	22	75	57.5	13.7	31	79	60.3	10.0	42	77	56.7	16.5	31	81	0.95
	BMI	24.1	4.5	18.4	33.9	23.5	4.3	16.4	31.9	23.7	4.2	16.2	40.0	24.5	3.8	15.6	30.4	0.76
Pain																		
	PainYRS	-	-	-	-	12.7	7.1	1.5	30	10.5	7.7	1	20	8.3	7.8	1	20	0.16
	PainNRS	-	-	-	-	5.5	2.9	0	10	3.2	2.8	0	9	3.7	1.9	0	7	0.01
	PSS	-	-	-	-	5.7	2.3	0	9	3.5	2.2	0	6.8	4.8	1.3	2.5	7.3	0.003
	PIS	-	-	-	-	5.5	2.6	0	9.3	4.3	2.9	0	8.3	4.8	2.0	0.9	8.3	0.33
Mood																		
	BDI_tot	7.5	6.9	0	24	18.8	10.4	4	45	17.8	14.7	0	48	16.1	8.8	0	30	0.002
	BDI_cogn	4.8	6.0	0	21	12.4	8.6	0	32	12.3	12.0	0	39	10.8	7.0	0	22	0.01
	BDI_som	2.8	1.8	0	6	6.4	2.6	3	13	5.5	3.2	0	9	5.3	2.7	0	11	<0.001
	STAI_S	29.5	5.8	20	44	41.0	9.5	28	68	38.0	15.9	20	76	41.8	14.2	27	72	<0.001
IA																		
	IAcc	0.6	0.2	0.2	1.0	0.3	0.3	0	1.0	0.4	0.3	0	1.0	0.4	0.3	0	1.0	0.01
	IAconf	59.1	16.4	26.0	90.7	31.9	29.3	0	82.7	32.7	29.0	0	84	36.0	30.2	0	92.7	0.01
MAIA																		
	NO	3.1	1.1	0.8	4.8	3.2	1.1	1.3	5	3.4	1.3	0.5	5	3.1	1.3	0	4.8	0.75
	ND	2.2	1.1	0.7	4.0	2.3	1.2	0	5	1.6	0.9	0.3	3.7	2.5	1.4	1	5	0.12
	NW	2.6	1.3	0	4.7	2.6	1.3	0	5	2.4	1.6	0	5.0	2.5	1.6	0	5	0.93
	AR	2.3	1.1	0.1	4.1	2.4	1.2	0.4	5	2.7	0.9	1.4	5.0	2.7	1.2	1.3	5	0.72
	EA	3.3	1.1	0.6	5.0	3.5	1.1	1.2	5	3.5	1.1	1.0	5.0	3.1	1.2	0.2	5	0.62
	SR	2.5	1.4	0	4.8	2.3	1.4	0	5	2.3	1.2	0	4.8	2.3	1.5	0.5	5	0.85
	BL	2.4	1.3	0	4.7	2.4	1.3	0.3	5	2.4	1.4	0.3	5.0	2.3	1.3	0.7	5	0.99
	TR	3.3	1.3	1.0	5.0	2.6	1.6	0	5	2.7	1.6	0	5.0	2.9	1.3	0	5	0.51

BMI: body mass index, PainYRS: pain duration in years, PainNRS: pain measured via numeric rating scale, PSS: BPI pain severity score, PIS: BPI pain interference score, BDI_tot: BDI total score, BDI_cogn: BDI cognitive factors, BDI_som: BDI somatic factors, STAI_S: STAI state anxiety, IAcc: interoceptive accuracy, IAconf: interoceptive confidence. MAIA subscales: MAIA_NO: Noticing, MAIA_ND: Not Distracting, MAIA_NW: Not Worrying, MAIA_AR: Attention Regulation, MAIA_EA: Emotional Awareness, MAIA_SR: Self-Regulation, MAIA_BL: Body Listening, MAIA_TR: Trusting.

**Table 3:** Pearson Correlation Matrix for principal variables in the CP patients (*N* = 60).

	Age	BMI	PainYrs	PainNRS	PSS	PSI	BDI	STAI_S	IAcc	IAconf
Age	—									
	—									
BMI	0.16	—								
	0.24	—								
PainYrs	0.05	−0.01	—							
	0.73	0.96	—							
PainNRS	−0.04	0.12	0.12	—						
	0.76	0.35	0.36	—						
PSS	−0.04	0.14	0.16	0.75 ***	—					
	0.78	0.29	0.22	<0.001	—					
PIS	−0.3 *	0.12	−0.03	0.53 ***	0.73 ***	—				
	0.02	0.37	0.85	<0.001	<0.001	—				
BDI	−0.13	0.16	−0.14	0.41 **	0.47 ***	0.63 ***	—			
	0.32	0.23	0.27	0.001	<0.001	<0.001	—			
STAI_S	−0.01	0.13	−0.03	0.37 **	0.38 **	0.44 ***	0.66 ***	—		
	0.94	0.35	0.81	0.004	0.003	<0.001	<0.001	—		
IAcc	0.1	−0.19	−0.07	0.07	−0.13	−0.15	−0.15	−0.28 *	—	
	0.46	0.15	0.58	0.62	0.34	0.27	0.26	0.03	—	
IAconf	0.19	−0.18	−0.14	−0.17	−0.16	−0.2	−0.24 *	−0.28 *	0.61 ***	—
	0.16	0.18	0.31	0.19	0.23	0.13	0.03	0.03	<0.001	—

* *p* < 0.05, ** *p* < 0.01, *** *p* < 0.001 BMI: body mass index, PainYRS: pain duration in years, PainNRS: pain measured via numeric rating scale, PSS: BPI pain severity score, PIS: BPI pain interference score, BDI: BDI total score, STAI_S: STAI state anxiety, IAcc: interoceptive accuracy, IAconf: interoceptive confidence.

**Table 4:** Psychiatric comorbidities and medication intake in CP sample.

	Frequency (*n*)	Percent (%)
*Psychiatric Comorbidities*		
Depression	17	28.33
Anxiety	4	6.66
*Medications*		
Anxiolitic	14	23.33
Antidepressant	37	61.67
Opiates	18	30.00
Non-opiates analgesic	18	30.00
Antiepilectic	31	51.67

## Supplementary Materials

The following are available online at https://www.mdpi.com/2075-4426/10/4/201/s1, Table S1: the specific pathologies of CP participants. Figure S1: Details of psychometric scores and demographic data for the experimental and the control group.
