# Supplementary material for: Altered Interoceptive Perception and the Effects of Interoceptive Analgesia in Musculoskeletal, Primary, and Neuropathic Chronic Pain Conditions
Source: J Pers Med. 2020 Oct 29;10(4):201. doi: 10.3390/jpm10040201 (PMC7712753; doi:10.3390/jpm10040201)
Supplement: Supplementary file 1 [file jpm-10-00201-s001.zip › Supplementary figure s1.pdf]

# Supplementary Material B

In the experimental condition, interoceptive tactile stimulation was delivered in fixed time intervals randomly presented in 6 blocks. Each block was composed of 6 stimuli of 8s, 10s, 12s, 14s, 16s, and 18s. Each stimulus was followed by a 6s pause. The entire duration of the stimulation was approximately 11 minutes. The identical procedure was followed for the control condition with static pressure stimuli. Fig.1

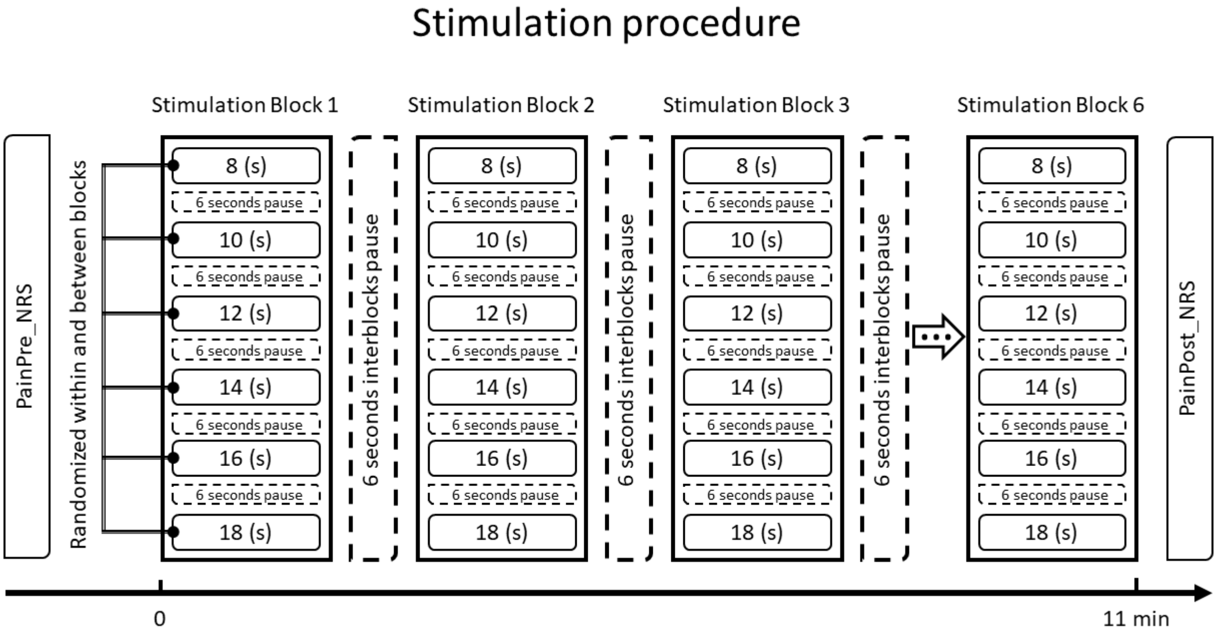


Figure. 1. Stimulation procedure.

## Study 2. Supplementary Statistics

Below are descriptive statistics for the chronic pain sample and the results of an ANOVAs that shows the effects of the interoceptive stimulation as a method for pain analgesia. 2x2 ANOVA with factors Time (pre and post-stimulation) and Condition (Experimental and Control). Additional analyses are provided controlling for relevant covariates: Age, Body Mass Index (BMI), Duration in years of the pathology (PainYrs), Depression (BDI), State Anxiety (STAI_S), Interoceptive Cardiac Accuracy (IAcc), Interoceptive Confidence (IAconf).

**Descriptive Statistics** : Control (0) and Experimental (1) group

**Descriptive Statistics**

| | Age | | BMI | | Pain_Yrs | | PainPre_NRS | | PainPost_NRS | | Pain_Reduction | |
|---|---|---|---|---|---|---|---|---|---|---|---|---|
| | 0 | 1 | 0 | 1 | 0 | 1 | 0 | 1 | 0 | 1 | 0 | 1 |
| **N** | 25 | 24 | 25 | 24 | 25 | 24 | 25 | 24 | 25 | 24 | 25 | 24 |
| **Mean** | 58.520 | 57.292 | 24.149 | 23.647 | 8.540 | 12.417 | 5.520 | 4.250 | 5.560 | 3.292 | 0.040 | -0.958 |
| **Std. Deviation** | 16.003 | 13.027 | 4.661 | 4.048 | 7.402 | 7.518 | 2.220 | 2.132 | 2.329 | 2.274 | 0.889 | 1.268 |
| **Minimum** | 31.000 | 31.000 | 15.623 | 16.233 | 1.000 | 1.000 | 1.000 | 2.000 | 1.000 | 0.000 | -3.000 | -3.000 |
| **Maximum** | 79.000 | 81.000 | 31.862 | 29.737 | 30.000 | 20.000 | 10.000 | 8.000 | 10.000 | 7.000 | 2.000 | 2.000 |

| | IAcc | | IAconf | | BDI | | STAI_S | |
|---|---|---|---|---|---|---|---|---|
| | 0 | 1 | 0 | 1 | 0 | 1 | 0 | 1 |
| **N** | 25 | 24 | 25 | 24 | 25 | 24 | 24 | 24 |
| **Mean** | 0.358 | 0.353 | 36.533 | 29.750 | 21.440 | 14.542 | 46.333 | 38.000 |
| **Std. Deviation** | 0.304 | 0.359 | 29.945 | 30.038 | 11.015 | 10.134 | 14.315 | 10.799 |
| **Minimum** | 0.000 | 0.000 | 0.000 | 0.000 | 3.000 | 0.000 | 28.000 | 20.000 |
| **Maximum** | 0.964 | 0.975 | 84.333 | 92.667 | 48.000 | 41.000 | 76.000 | 68.000 |

**Frequencies for Pain_rcd: 1 SMP, 2 PP, 3 NP**

| Condition | Pain_rcd | Frequency | Percent | Valid Percent | Cumulative Percent |
|---|---|---|---|---|---|
| 0 | 1 | 5 | 20.000 | 20.000 | 20.000 |
| | 2 | 10 | 40.000 | 40.000 | 60.000 |
| | 3 | 10 | 40.000 | 40.000 | 100.000 |
| | Total | 25 | 100.000 | | |
| 1 | 1 | 8 | 33.333 | 33.333 | 33.333 |
| | 2 | 9 | 37.500 | 37.500 | 70.833 |
| | 3 | 7 | 29.167 | 29.167 | 100.000 |
| | Total | 24 | 100.000 | | |

*SMP: secondary musculoskeletal pain, PP: primary pain, NP: neuropathic pain*

**Supplementary analyses.**
To evaluate the effect of the stimulation on chronic pain, we performed an RM ANOVA with factor Time (PainPre_NRS and PainPost_NRS) and factor group (Control = 0 and Experimental = 1). Assumptions were satisfied. Post-hoc comparisons used Bonferroni correction to evaluate significant results. Furthermore, to investigate the effect of possible confounding variables, we performed several additional RM ANOVAs controlling for all relevant covariates such as age, BMI, IAcc, IAconf, BDI, and STAI_S. Calculations were performed in Jasp 0.11.

**Results Study 2**
*Pain reduction after interoceptive tactile stimulation*
ANOVA showed a significant main effect of Time [$F$ (1, 47) = 8.68, $p = 0.005$, $\eta_p^2 = 0.156$]. More importantly, the interaction effect of Time x Condition was highly significant [$F(1,47) = 10.26$, $p = 0.002$, $\eta_p^2 = 0.18$]. Post hoc analyses, with Bonferroni correction, indicated that the main effect was driven entirely by the Experimental group who received the interoceptive stimulation. They reported a significant reduction in pain [Mean Difference = -0.96, SE = 0.22, t = - 4.30, $p_b < .001$, Cohen's d = -0.61] between before [Mean = 4.25, SD = 2.13] and after stimulation [Mean = 3.29, SD = 2.27]. By contrast, no significant difference in pain [Mean Difference = 0.04, SE = 0.22, t = 0.183, pb = 1.00, Cohen's d = 0.03] was reported in the Control group between before [Mean = 5.52, SD = 2.22] and after the stimulation [Mean = 5.56, SD = 2.33]. No significant difference was found in pain at the baseline between the Control and Experimental groups [Mean Difference = 1.27, SE = 0.64, t = 1.984, $p_b = 0.315$, Cohen's d = 0.283]. These results are illustrated in Fig. 6.
We performed several additional RM ANOVAs to assess the effects of relevant covariates. The results remained significant after controlling for the following covariates: demographics [Age p = 0.23, BMI p = 0.43]; mood [BDI p = 0.87, STAI_S p = 0.26]. Interestingly, neither of the interoceptive variables [IAc p = 0.96, IAw p = 0.68] had any significant effect on pain reduction

***<u>Repeated Measures ANOVA</u>***
**Within Subjects Effects**

| | Sphericity Correction | Sum of Squares | df | Mean Square | F | p | VS-MPR* | η² | η² p | η² G | ω² |
|---|---|---|---|---|---|---|---|---|---|---|---|
| Time | None | 5.163 | 1.000 | 5.163 | 8.680 | 0.005 | 13.902 | 0.009 | 0.156 | 0.011 | 0.009 |
| | Greenhouse-Geisser | 5.163 | 1.000 | 5.163 | 8.680 | 0.005 | 13.902 | 0.009 | 0.156 | 0.011 | 0.009 |
| | Huynh-Feldt | 5.163 | 1.000 | 5.163 | 8.680 | 0.005 | 13.902 | 0.009 | 0.156 | 0.011 | 0.009 |
| Time ✻ Condition | None | 6.102 | 1.000 | 6.102 | 10.258 | 0.002 | 25.027 | 0.011 | 0.179 | 0.013 | 0.011 |
| | Greenhouse-Geisser | 6.102 | 1.000 | 6.102 | 10.258 | 0.002 | 25.027 | 0.011 | 0.179 | 0.013 | 0.011 |
| | Huynh-Feldt | 6.102 | 1.000 | 6.102 | 10.258 | 0.002 | 25.027 | 0.011 | 0.179 | 0.013 | 0.011 |
| Residual | None | 27.959 | 47.000 | 0.595 | | | | | | | |

**Within Subjects Effects**

| | Sphericity Correction | Sum of Squares | df | Mean Square | F | p | VS-MPR* | η² | η² p | η² G | ω² |
|---|---|---|---|---|---|---|---|---|---|---|---|
| | Greenhouse-Geisser | 27.959 | 47.000 | 0.595 | | | | | | | |
| | Huynh-Feldt | 27.959 | 47.000 | 0.595 | | | | | | | |

*Note.* Type III Sum of Squares

* Vovk-Sellke Maximum *p* -Ratio: Based the *p* -value, the maximum possible odds in favor of $H_1$ over $H_0$ equals 1/(-e *p* log( *p* )) for *p* ≤ .37 (Sellke, Bayarri, & Berger, 2001).

**Between Subjects Effects**

| | Sum of Squares | df | Mean Square | F | p | VS-MPR* | η² | η² p | η² G | ω² |
|---|---|---|---|---|---|---|---|---|---|---|
| Condition | 76.652 | 1 | 76.652 | 8.116 | 0.006 | 11.250 | 0.147 | 0.147 | 0.140 | 0.127 |
| Residual | 443.899 | 47 | 9.445 | | | | | | | |

*Note.* Type III Sum of Squares

* Vovk-Sellke Maximum *p* -Ratio: Based the *p* -value, the maximum possible odds in favor of $H_1$ over $H_0$ equals 1/(-e *p* log( *p* )) for *p* ≤ .37 (Sellke, Bayarri, & Berger, 2001).

### *1.1.1 Assumption Checks*

**Test of Sphericity**

| | Mauchly's W | Approx. X² | df | p | VS-MPR | Greenhouse-Geisser ε | Huynh-Feldt ε | Lower Bound ε |
|---|---|---|---|---|---|---|---|---|
| Time | 1.000 [a] | NaN [a] | NaN [a] | NaN [a] | 1.000 | 1.000 [a] | 1.000 [a] | 1.000 [a] |

[a] Singular error SSP matrix: The repeated measure has only two levels, or more levels than observations. When the repeated measure has two levels, the assumption of sphericity is always met.

**Test for Equality of Variances (Levene's)**

| | F | df1 | df2 | p | VS-MPR |
|---|---|---|---|---|---|
| PainPre_NRS | 0.137 | 1.000 | 47.000 | 0.713 | 1.000 |
| PainPost_NRS | 0.090 | 1.000 | 47.000 | 0.766 | 1.000 |

### *1.1.2 Post Hoc Tests*

**Post Hoc Comparisons - Time ✻ Condition**

| | | | 95% CI of Mean Difference | | | | | | |
|---|---|---|---|---|---|---|---|---|---|
| | | Mean Difference | Lower | Upper | SE | t | Cohen's d | p bonf | p tukey |
| Post,0 | Post,1 | 2.268 | 0.513 | 4.023 | 0.640 | 3.543 | 0.506 | 0.005 | 0.005 |
| | Pre,0 | 0.040 | -0.561 | 0.641 | 0.218 | 0.183 | 0.026 | 1.000 | 0.998 |
| | Pre,1 | 1.310 | -0.445 | 3.065 | 0.640 | 2.046 | 0.292 | 0.274 | 0.184 |
| Post,1 | Pre,0 | -2.228 | -3.983 | -0.473 | 0.640 | -3.480 | -0.497 | 0.006 | 0.005 |
| | Pre,1 | -0.958 | -1.572 | -0.345 | 0.223 | -4.304 | -0.615 | < .001 | < .001 |
| Pre,0 | Pre,1 | 1.270 | -0.485 | 3.025 | 0.640 | 1.984 | 0.283 | 0.315 | 0.207 |

*Note.* Cohen's d does not correct for multiple comparisons.

*Note.* Bonferroni adjusted confidence intervals.

## ***Repeated Measures ANOVA / factor clinical cluster***

**Within Subjects Effects**

| | Sum of Squares | df | Mean Square | F | p | η² | η² p |
|---|---|---|---|---|---|---|---|
| Time | 4.593 | 1 | 4.593 | 7.332 | 0.010 | 0.008 | 0.146 |
| Time ✻ Condition | 6.069 | 1 | 6.069 | 9.688 | 0.003 | 0.011 | 0.184 |
| Time ✻ Clinical_Cluster | 0.533 | 2 | 0.267 | 0.426 | 0.656 | 0.001 | 0.019 |

**Within Subjects Effects**

| | Sum of Squares | df | Mean Square | F | p | η² | η² p |
|---|---|---|---|---|---|---|---|
| Time ✻ Condition ✻ Clinical_Cluster | 0.407 | 2 | 0.204 | 0.325 | 0.724 | 0.001 | 0.015 |
| Residual | 26.937 | 43 | 0.626 | | | | |

*Note.* Type III Sum of Squares

**Between Subjects Effects**

| | Sum of Squares | df | Mean Square | F | p | η² | η² p |
|---|---|---|---|---|---|---|---|
| Condition | 82.082 | 1 | 82.082 | 10.766 | 0.002 | 0.160 | 0.200 |
| Clinical_Cluster | 80.290 | 2 | 40.145 | 5.265 | 0.009 | 0.156 | 0.197 |
| Condition ✻ Clinical_Cluster | 23.509 | 2 | 11.755 | 1.542 | 0.226 | 0.046 | 0.067 |
| Residual | 327.837 | 43 | 7.624 | | | | |

*Note.* Type III Sum of Squares

## 2 Assumption Checks

**Test for Equality of Variances (Levene's)**

| | F | df1 | df2 | p |
|---|---|---|---|---|
| PainPre_NRS | 0.657 | 5.000 | 43.000 | 0.658 |
| PainPost_NRS | 1.034 | 5.000 | 43.000 | 0.410 |

## 3 Post Hoc Tests

**Post Hoc Comparisons - Time ✻ Condition**

| | | Mean Difference | SE | t | p bonf |
|---|---|---|---|---|---|
| Post,0 | Post,1 | 2.397 | 0.598 | 4.012 | 0.001 |
| | Pre,0 | 0.067 | 0.236 | 0.283 | 1.000 |
| | Pre,1 | 1.439 | 0.598 | 2.408 | 0.119 |
| Post,1 | Pre,0 | -2.331 | 0.598 | -3.900 | 0.002 |
| | Pre,1 | -0.958 | 0.230 | -4.172 | < .001 |
| Pre,0 | Pre,1 | 1.372 | 0.598 | 2.296 | 0.155 |

## 4 Descriptives

**Descriptives: 1 SMP, 2 PP, 3 NP**

| Time | Condition | Clinical_Cluster | Mean | SD | N |
|---|---|---|---|---|---|
| Pre | 0 | 1 | 5.800 | 2.387 | 5 |
| | | 2 | 7.000 | 1.563 | 10 |
| | | 3 | 3.900 | 1.663 | 10 |
| | 1 | 1 | 3.250 | 2.053 | 8 |
| | | 2 | 5.333 | 2.121 | 9 |
| | | 3 | 4.000 | 1.826 | 7 |
| Post | 0 | 1 | 6.000 | 3.082 | 5 |
| | | 2 | 6.700 | 1.636 | 10 |
| | | 3 | 4.200 | 1.989 | 10 |
| | 1 | 1 | 2.375 | 2.669 | 8 |
| | | 2 | 4.333 | 2.121 | 9 |
| | | 3 | 3.000 | 1.633 | 7 |

***Repeated Measures ANOVA / Covariate BDI***

**Within Subjects Effects**

| | Sum of Squares | df | Mean Square | F | p | VS-MPR* | η² | η² p | η² G | ω² |
|---|---|---|---|---|---|---|---|---|---|---|
| Time | 1.550 | 1 | 1.550 | 2.552 | 0.117 | 1.466 | 0.003 | 0.053 | 0.004 | 0.002 |
| Time ✻ Condition | 5.309 | 1 | 5.309 | 8.740 | 0.005 | 14.120 | 0.010 | 0.160 | 0.012 | 0.011 |
| Time ✻ BDI | 0.016 | 1 | 0.016 | 0.026 | 0.872 | 1.000 | 0.000 | 0.001 | 0.000 | 0.000 |
| Residual | 27.943 | 46 | 0.607 | | | | | | | |

**Within Subjects Effects**

| | Sum of Squares | df | Mean Square | F | p | VS-MPR* | $\eta^2$ | $\eta^2_p$ | $\eta^2_G$ | $\omega^2$ |
|---|---|---|---|---|---|---|---|---|---|---|

*Note.* Type III Sum of Squares

* Vovk-Sellke Maximum *p* -Ratio: Based the *p* -value, the maximum possible odds in favor of $H_1$ over $H_0$ equals 1/(-e *p* log( *p* )) for *p* ≤ .37 (Sellke, Bayarri, & Berger, 2001).

***Repeated Measures ANOVA / Covariate STAI_S***

**Within Subjects Effects**

| | Sum of Squares | df | Mean Square | F | p | VS-MPR* | $\eta^2$ | $\eta^2_p$ | $\eta^2_G$ | $\omega^2$ |
|---|---|---|---|---|---|---|---|---|---|---|
| Time | 2.224 | 1 | 2.224 | 3.684 | 0.061 | 2.150 | 0.005 | 0.076 | 0.005 | 0.004 |
| Time ✻ Condition | 4.153 | 1 | 4.153 | 6.880 | 0.012 | 6.997 | 0.008 | 0.133 | 0.010 | 0.009 |
| Time ✻ STAI_S | 0.798 | 1 | 0.798 | 1.323 | 0.256 | 1.054 | 0.002 | 0.029 | 0.002 | 0.000 |
| Residual | 27.160 | 45 | 0.604 | | | | | | | |

*Note.* Type III Sum of Squares

* Vovk-Sellke Maximum *p* -Ratio: Based the *p* -value, the maximum possible odds in favor of $H_1$ over $H_0$ equals 1/(-e *p* log( *p* )) for *p* ≤ .37 (Sellke, Bayarri, & Berger, 2001).

***Repeated Measures ANOVA / Covariate IAcc***

**Within Subjects Effects**

| | Sum of Squares | df | Mean Square | F | p | VS-MPR* | $\eta^2$ | $\eta^2_p$ | $\eta^2_G$ | $\omega^2$ |
|---|---|---|---|---|---|---|---|---|---|---|
| Time | 2.446 | 1 | 2.446 | 4.024 | 0.051 | 2.432 | 0.004 | 0.080 | 0.005 | 0.004 |
| Time ✻ Condition | 6.100 | 1 | 6.100 | 10.037 | 0.003 | 22.856 | 0.011 | 0.179 | 0.013 | 0.011 |
| Time ✻ IAcc | 0.002 | 1 | 0.002 | 0.003 | 0.958 | 1.000 | 0.000 | 0.000 | 0.000 | 0.000 |
| Residual | 27.957 | 46 | 0.608 | | | | | | | |

*Note.* Type III Sum of Squares

* Vovk-Sellke Maximum *p* -Ratio: Based the *p* -value, the maximum possible odds in favor of $H_1$ over $H_0$ equals 1/(-e *p* log( *p* )) for *p* ≤ .37 (Sellke, Bayarri, & Berger, 2001).

***Repeated Measures ANOVA / Covariate IAconf***

**Within Subjects Effects**

| | Sum of Squares | df | Mean Square | F | p | VS-MPR* | $\eta^2$ | $\eta^2_p$ | $\eta^2_G$ | $\omega^2$ |
|---|---|---|---|---|---|---|---|---|---|---|
| Time | 1.604 | 1 | 1.604 | 2.648 | 0.110 | 1.511 | 0.003 | 0.054 | 0.004 | 0.002 |
| Time ✻ Condition | 6.204 | 1 | 6.204 | 10.246 | 0.002 | 24.686 | 0.011 | 0.182 | 0.014 | 0.012 |
| Time ✻ IAconf | 0.104 | 1 | 0.104 | 0.171 | 0.681 | 1.000 | 0.000 | 0.004 | 0.000 | 0.000 |
| Residual | 27.856 | 46 | 0.606 | | | | | | | |

*Note.* Type III Sum of Squares

* Vovk-Sellke Maximum *p* -Ratio: Based the *p* -value, the maximum possible odds in favor of $H_1$ over $H_0$ equals 1/(-e *p* log( *p* )) for *p* ≤ .37 (Sellke, Bayarri, & Berger, 2001).

***Repeated Measures ANOVA / Covariate BMI***

**Within Subjects Effects**

| | Sum of Squares | df | Mean Square | F | p | VS-MPR* | $\eta^2$ | $\eta^2_p$ | $\eta^2_G$ | $\omega^2$ |
|---|---|---|---|---|---|---|---|---|---|---|
| Time | 1.008 | 1 | 1.008 | 1.682 | 0.201 | 1.140 | 0.002 | 0.035 | 0.002 | 0.001 |
| Time ✻ Condition | 5.906 | 1 | 5.906 | 9.849 | 0.003 | 21.319 | 0.011 | 0.176 | 0.012 | 0.011 |
| Time ✻ BMI | 0.376 | 1 | 0.376 | 0.627 | 0.433 | 1.000 | 0.001 | 0.013 | 0.001 | 0.000 |
| Residual | 27.583 | 46 | 0.600 | | | | | | | |

*Note.* Type III Sum of Squares

* Vovk-Sellke Maximum *p* -Ratio: Based the *p* -value, the maximum possible odds in favor of $H_1$ over $H_0$ equals 1/(-e *p* log( *p* )) for *p* ≤ .37 (Sellke, Bayarri, & Berger, 2001).

***Repeated Measures ANOVA  / Covariate Age***

**Within Subjects Effects**

| | Sum of Squares | df | Mean Square | F | p | VS-MPR* | $\eta^2$ | $\eta^2_p$ | $\eta^2_G$ | $\omega^2$ |
|---|---|---|---|---|---|---|---|---|---|---|
| Time | 2.085 | 1 | 2.085 | 3.539 | 0.066 | 2.045 | 0.004 | 0.071 | 0.004 | 0.003 |
| Time ✻ Condition | 5.897 | 1 | 5.897 | 10.008 | 0.003 | 22.614 | 0.011 | 0.179 | 0.012 | 0.011 |
| Time ✻ Age | 0.856 | 1 | 0.856 | 1.453 | 0.234 | 1.082 | 0.002 | 0.031 | 0.002 | 0.001 |
| Residual | 27.103 | 46 | 0.589 | | | | | | | |

*Note.* Type III Sum of Squares

* Vovk-Sellke Maximum *p* -Ratio: Based the *p* -value, the maximum possible odds in favor of $H_1$ over $H_0$ equals 1/(-e *p* log( *p* )) for $p \leq .37$ (Sellke, Bayarri, & Berger, 2001).

***Repeated Measures ANOVA / Covariate Pain Yrs***

**Within Subjects Effects**

| | Sum of Squares | df | Mean Square | F | p | VS-MPR* | $\eta^2$ | $\eta^2_p$ | $\eta^2_G$ | $\omega^2$ |
|---|---|---|---|---|---|---|---|---|---|---|
| Time | 0.356 | 1 | 0.356 | 0.602 | 0.442 | 1.000 | 0.001 | 0.013 | 0.001 | 0.000 |
| Time ✻ Condition | 4.700 | 1 | 4.700 | 7.941 | 0.007 | 10.473 | 0.008 | 0.147 | 0.011 | 0.009 |
| Time ✻ Pain_Yrs | 0.734 | 1 | 0.734 | 1.240 | 0.271 | 1.040 | 0.001 | 0.026 | 0.002 | 0.000 |
| Residual | 27.225 | 46 | 0.592 | | | | | | | |

*Note.* Type III Sum of Squares

* Vovk-Sellke Maximum *p* -Ratio: Based the *p* -value, the maximum possible odds in favor of $H_1$ over $H_0$ equals 1/(-e *p* log( *p* )) for $p \leq .37$ (Sellke, Bayarri, & Berger, 2001).
