# Supplementary material for: Altered Interoceptive Perception and the Effects of Interoceptive Analgesia in Musculoskeletal, Primary, and Neuropathic Chronic Pain Conditions
Source: J Pers Med. 2020 Oct 29;10(4):201. doi: 10.3390/jpm10040201 (PMC7712753; doi:10.3390/jpm10040201)
Supplement: Supplementary file 1 [file jpm-10-00201-s001.zip › Supplementary table s1.pdf]

Supplementary Material A – Table A1

Age	Sex	BMI	Arm	Psychological Diagnosis	CP Diagnosis	CP Cluster	CP recod	PainYRS	PainNRS	Rescue medications	Prescribed medications
61	F	19.05	Dx	Depression	Arthritis	Chronic secondary musculoskeletal pain	1	12	2	NO	YES
50	F	20.02	Dx		Osteoarthrosis	Chronic secondary musculoskeletal pain	1	12	2	NO	NO
42	F	24.97	Dx	Depression	Arthritis	Chronic secondary musculoskeletal pain	1	20	6	NO	YES
53	F	24.09	Dx		Paget syndromes	Chronic secondary musculoskeletal pain	1	10	0	NO	YES
53	F	26.98	Dx		Fibromyalgia	Chronic primary pain	2	10	9	NO	YES
52	F	29.73	Dx	Depression	Arthritis	Chronic secondary musculoskeletal pain	1	8	3	NO	YES
62	M	26.36	Dx		Arthritis	Chronic secondary musculoskeletal pain	1	20	6	NO	YES
54	F	24.46	Dx	Depression	Fibromyalgia	Chronic primary pain	2	3	2	NO	YES
43	F	24.67	Dx		Fibromyalgia	Chronic primary pain	2	15	7	NO	YES
51	F	23.42	Dx		Arthritis	Chronic secondary musculoskeletal pain	1	20	0	NO	YES
57	F	18.82	Dx		Fibromyalgia	Chronic primary pain	2	20	6	NO	YES
66	F	19.11	Dx		Arthritis	Chronic secondary musculoskeletal pain	1	20	7	NO	YES
34	F	19.48	Dx		Fibromyalgia	Chronic primary pain	2	8	7	NO	NO
69	F	23.53	Dx		Fibromyalgia	Chronic primary pain	2	20	0	NO	YES
50	F	16.23	Dx		Arthritis	Chronic secondary musculoskeletal pain	1	20	2	NO	YES
56	F	21.63	Dx		Arthritis	Chronic secondary musculoskeletal pain	1	15	0	NO	YES
56	F	20.95	Dx	Depression	Traumatic rib injury	Chronic secondary musculoskeletal pain	1	1	2	NO	YES
66	F	26.03	Dx	Depression	PN, Polyneuropathy, DNP	Chronic neuropathic pain	3	20	3	NO	YES
51	F	27.58	Dx		PN, PNI, PRNP	Chronic neuropathic pain	3	20	6	NO	YES
78	M	25.46	Dx		PN, Polyneuropathy, DNP	Chronic neuropathic pain	3	1	3	NO	YES
74	F	28.04	Dx	Depression	Chronic tension-type headache	Chronic primary pain	2	15	7	NO	YES
59	F	19.31	Dx	Depression	Fibromyalgia	Chronic primary pain	2	20	4	NO	YES
57	F	29.06	Dx		Fibromyalgia	Chronic primary pain	2	8	3	NO	YES
68	F	27.76	Dx		CNP, syringomyelia, DNP	Chronic neuropathic pain	3	20	7	NO	YES
72	F	29.72	Dx		Low back pain/spondylosis	Chronic secondary musculoskeletal pain	1	2	2	NO	YES

60	M	26.19	Dx		Osteoarthrosis	Chronic secondary musculoskeletal pain	1	20	0	NO	YES
49	F	23.38	Dx	Depression	Fibromyalgia	Chronic primary pain	2	2	8	NO	YES
31	F	21.45	Dx		Fibromyalgia	Chronic primary pain	2	15	4	NO	YES
72	F	17.74	Dx		Low back pain/spondylosis	Chronic secondary musculoskeletal pain	1	3	9	NO	YES
49	F	17.99	Dx		Chronic primary pelvic pain	Chronic primary pain	2	2	0	NO	YES
45	M	19.55	Dx		PN, CR, DNP	Chronic neuropathic pain	3	2	2	NO	YES
71	F	24.65	Dx	Depression	PN, PNI, PRNP	Chronic neuropathic pain	3	1	0	NO	YES
67	F	23.23	Dx		Low back pain/spondylosis	Chronic secondary musculoskeletal pain	1	5	4	NO	YES
70	F	16.44	Dx		Fibromyalgia	Chronic primary pain	2	10	6	NO	YES
58	M	21.46	Dx	Depression	Fibromyalgia	Chronic primary pain	2	10	0	NO	YES
71	M	24.09	Dx		CNP, syringomyelia, DNP	Chronic neuropathic pain	3	20	2	NO	YES
81	M	20.32	Dx		PN, PHN, DNP	Chronic neuropathic pain	3	15	4	NO	YES
53	M	27.73	Dx		PN, PNI, DNP	Chronic neuropathic pain	3	1	3	NO	YES
76	F	28.62	Dx		Osteoarthrosis	Chronic secondary musculoskeletal pain	1	2	6	NO	YES
43	F	28.04	Dx	Depression	PN, PNI, DNP	Chronic neuropathic pain	3	4	5	NO	YES
71	F	18.09	Dx		Chronic primary pelvic pain	Chronic primary pain	2	8	8	NO	YES
77	F	25.16	Dx		PN, PNI, PRNP	Chronic neuropathic pain	3	2	1	NO	YES
49	M	25.08	Dx		PN, PNI, DNP	Chronic neuropathic pain	3	1	4	NO	YES
44	M	25.14	Dx		PN, PNI, DNP	Chronic neuropathic pain	3	3	4	NO	YES
31	F	26.17	Dx	.	PN, PNI, DNP	Chronic neuropathic pain	3	3	6	NO	YES
77	F	25.86	Dx	Depression and Generalized anxiety disorder	Low Back Pain/spondylosis	Chronic secondary musculoskeletal pain	1	2	3	Metamizol (Antidol)	YES
53	F	29.96	Dx	Depression Generalized anxiety disorder	Low Back Pain/spondylosis	Chronic secondary musculoskeletal pain	1	5	7	NO	YES
75	M	26.72	Dx		Fibromyalgia	Chronic primary pain	2	8	5	NO	YES
79	F	19.95	Dx		Fibromyalgia	Chronic primary pain	2	12	7	NO	YES
76	M	26.64	Dx	Generalized anxiety disorder	Fibromyalgia	Chronic primary pain	2	30	5	NO	YES
52	F	30.11	Dx	Depression	Fibromyalgia	Chronic primary pain	2	20	8	NO	YES
44	F	23.52	Sx		PN, polyneuropathy, DNP	Chronic neuropathic pain	3	3	6	NO	YES
69	F	23.76	Dx		Osteoarthrosis	Chronic secondary musculoskeletal pain	1	3	0	NO	YES

44	F	25.43	Dx		Fibromyalgia	Chronic primary pain	2	7	7	NO	YES
31	F	15.62	Dx		CNP, SCI, DNP	Chronic neuropathic pain	3	10	3	NO	YES
44	F	18.40	Dx		PN, CR, DNP	Chronic neuropathic pain	3	9	3	NO	YES
71	F	24.91	Dx	Depression	Fibromyalgia	Chronic primary pain	2	10	10	NO	YES
74	M	30.43	Dx	Depression and Generalized anxiety disorder	PN, PNI, PRNP	Chronic neuropathic pain	3	15	5	NO	YES
42	F	20.95	Dx		Fibromyalgia	Chronic primary pain	2	20	8	NO	YES
56	F	31.86	Dx	Depression	Fibromyalgia	Chronic primary pain	2	20	6	NO	YES

TABLE 1. Key: Peripheral neuropathic pain (PN), central neuropathic pain (CNP), peripheral nerve injury (PNI) spinal cord injury (SCI), cervical radiculopathy (CR), postherpetic neuralgia (PHN), Possible neuropathic pain (PNP), probable neuropathic pain (PRNP), definite neuropathic pain (DNP).
CP patients were asked to continue their prescribed medication according to the normal schedule and to avoid pain rescue medications in the 8 hours before the experiment. 'Prescribed Medications' indicates if the patient continued his or her prescribed medication. 'Rescued Medications' reports if the patient used a pain rescue medication in the 8 hours before the experiment.
